# Exploring Barriers to Timely Diagnosis and Treatment of Tuberculosis in Selangor, Malaysia: A Qualitative Study

**DOI:** 10.4269/ajtmh.24-0224

**Published:** 2024-10-01

**Authors:** Punitha Makeswaran, Shamsul Azhar Shah, Nazarudin Safian

**Affiliations:** Department of Public Health Medicine, Faculty of Medicine, Universiti Kebangsaan Malaysia, Kuala Lumpur, Malaysia

## Abstract

Tuberculosis (TB) occurs as one of the highest in the state of Selangor, Malaysia and its causes are currently underexplored especially from patients’ perspectives of the disease. This study assesses perspectives from patients in relations to determinants of delayed presentation, diagnosis, and treatment of TB. The study utilises a qualitative methodology involving focus group discussions and in-depth interviews with patients selected based on specific inclusion and exclusion criteria. The research covered components which include social demographic, sociocultural factors, health-seeking behaviors, social support and resources, previous knowledge and experience as a TB patient, and treatment pathway. Thematic analysis was performed using NVivo 12 analysis software to interpret the data. This study aims to identify key barriers and facilitators of prompt presentation and diagnosis of TB patients. Results revealed that socioeconomic status, challenges faced during TB diagnosis and treatment, knowledge of TB, patient and healthcare-related factors, and health-seeking behaviours were the main emerging themes. Improvements are required in the areas of access to healthcare facilities, minimizing cost, providing specific clinics and proximity of health facilities to facilitate a prompt response, increasing capacity to isolate infected persons, and provision of adequate treatment. It is revealed that financial constraints, inaccessibility and long distance to healthcare facilities, poor knowledge of TB, and lack of family and social support contributed to delayed TB presentation and diagnosis. Findings from this study can be utilized to develop effective, locally tailored interventions to address delayed presentation and diagnosis of TB patients in the nation.

## INTRODUCTION

Tuberculosis (TB) is an airborne communicable disease caused by *Mycobacterium tuberculosis*, an anaerobic pathogenic bacillus.^1^^,^^2^ Tuberculosis constitutes a major threat to global health and well-being.^1^^,^^3^ Although early diagnosis and prompt treatment are pertinent for effective TB control, a report in 2018 revealed that approximately 3 million TB cases were undiagnosed, especially in low- and middle-income countries.^2^^,^^3^ These estimates are more likely to be exacerbated given the effects of COVID-19 on healthcare delivery, care seeking, and diagnosis of TB.^2^^,^^3^

Diagnostic delay refers to a delay in the duration between symptom onset and treatment.^4^ Tuberculosis patients with diagnostic delay face a higher risk of experiencing severe disease status during presentation.^4^ Meanwhile, a healthcare system delay reflects a delay between the first visit to a healthcare facility and the beginning of treatment. Delay in diagnosis and commencement of treatment may impair treatment outcomes and increase the risk of TB transmission in the community, thereby constituting a significant barrier to TB elimination.^4^ A systematic review found that almost half of TB patients delayed seeking care for at least 1 month, with a median diagnostic delay ranging from 30 to 367 days.^5^ This delay is either patient or healthcare related, with the former attributed to challenges in accessing healthcare or stigma,^6^ whereas barriers such as inadequate testing infrastructure and poor TB screening guidelines are the predominant healthcare-related factors.^7^^,^^8^

Diagnostic delay is a multifactorial problem in TB management with factors such as pulmonary comorbidities, HIV infection, poor access to care, poverty, self-medication practices, low awareness of TB, stigma, and history of immigration playing significant roles.^6^^,^^7^ Overall, accumulated evidence from recent systematic reviews revealed that socioeconomic, demographic, and health-related factors are associated with delayed diagnosis and treatment of TB patients.^4^^,^^5^ Diagnostic delay may also stem from events related to the clinician such as low clinical suspicion, time constraints, lack of continuity of patient care, and poor clinician-patient communication.^8^ However, the strength of these associations is shaped by the TB burden in different countries and regions.

Tuberculosis is an endemic disease in Malaysia. With a notification rate of less than 100 cases for every 100,000 people, Malaysia is currently categorized as an intermediate TB-burden country.^9^ Tuberculosis is also notifiable by law in Malaysia, as stipulated in the Laws of Malaysia Act 342 – under the Prevention and Control of Infectious Diseases. About 91% of TB cases in Malaysia are pulmonary TB.^9^ To address the TB burden, the Malaysian government has implemented diverse strategies aligning with the WHO’s End TB Strategy goals. Examples include collaboration between the National HIV/AIDS Control Program and the National Tuberculosis Control Program, aimed at adjusting guidelines and case management protocols for TB.^10^

Nonetheless, notified TB cases continue to rise in Malaysia.^10^^,^^11^ Incomplete treatment, a high default rate, and the presence of resistant strains are among the factors that perpetuate persistent TB transmission in the community.^6^ Events such as loss to follow-up and poor medication adherence have also contributed significantly to poor treatment outcomes.^12^^–^^16^ Accumulated evidence from studies conducted in Malaysia reflects that patient-related factors constitute the main challenge to TB management and control strategies. A few studies conducted in Sabah and Sarawak revealed that the duration between the onset of major symptoms and the first medical consultation was 30 days, and delayed health-seeking behavior and limited access to TB care accounted for 20–30% of total TB cases.^16^^,^^17^

In relation to patient factors, involving those who are most affected by TB in research and intervention programs has been increasingly recognized as a strategy to ensure equitable and culturally appropriate access to care.^18^^,^^19^ Nevertheless, there is a dearth of information on the feasibility and progress of existing programs. This research gap can be bridged by understanding the lived experiences and realities of TB patients and the general public, who constitute the main targets of TB management programs.^20^^,^^21^

Despite the TB burden of Selangor topping the list in Malaysia, there are few published data on diagnostic and healthcare system delays, particularly from the lived experiences of TB patients in the country. Elucidating real and perceived barriers faced by TB patients and other key stakeholders is pertinent to developing effective interventions and addressing the identified issues. This study aimed to assess patients’ perspectives regarding the determinants of delayed presentation, diagnosis, and treatment of TB among patients in Selangor, Malaysia.

## MATERIALS AND METHODS

### Study area and study design.

This study was conducted in Selangor, the state with the highest TB burden in Malaysia.^18^^,^^19^ All the health clinics and specialized TB treatment centers in all districts in Selangor were considered in this study. The study design entailed a qualitative approach by conducting a focus group discussion (FGD) with the study population, comprising patients who had confirmed TB and visited any of the health facilities in Selangor, Malaysia.

### Eligibility criteria.

The inclusion criteria entailed TB patients with smear-positive pulmonary tuberculosis results who were 18 years old or above. In addition, the patient had to reside in the study location, be currently visiting one of the health facilities in Selangor for TB care and management, and have already completed the 6-month course of treatment. These inclusion criteria were considered to recruit participants with good knowledge of TB and the capacity to share their lived experiences as TB patients. Those who were unable to provide consent and did not fulfill the aforementioned criteria were excluded.

### Sampling and research participants.

Purposive and snowball sampling techniques were used in this study. Because this study aimed to recruit TB patients with the characteristics mentioned earlier in the eligibility criteria, purposive sampling was considered appropriate. Thus, the first step was to seek the approval of either the director general or health officer in the three health facilities for TB management located in Selangor. Thereafter, the principal researcher was instructed to reach out physically to potential TB patients visiting the various healthcare centers. A total of 25 TB patients were contacted and briefed about the research objectives and procedures and asked for their consent to participate in the FGDs. Overall, 10 of them agreed to participate in the study, and their informed consent was recorded by signing the consent form. The remaining 15 patients declined participation either owing to busy schedules or being too shy to speak or without giving any reason.

### Focus group discussion and in-depth interviews.

A semi-structured questionnaire was developed from a highly cited WHO survey of diagnostic and treatment delays in TB patients.^22^ The FGD and interview form were used to collect qualitative data on the factors associated with delayed presentation, diagnosis, and treatment of TB patients in Selangor, Malaysia. The discussion was guided to encourage the participants to share their thoughts and opinions on the research area. The session was conducted in person with all the TB patients who agreed to participate a week before the scheduled date. The FGD and in-depth interview were performed on November 20, 2021. The interview started with the session entailing an introductory remark. During this period, the interviewer welcomed the participants and explained the purpose of the session, as well as the study protocol and several dos and don’ts during the session.

Six components were covered during the FGD as follows: social demographic information, sociocultural factors, health-seeking behaviors, social support and resources, previous knowledge and experience as a TB patient, and treatment pathway. Each component was executed by asking some of the following questions: Can you briefly introduce yourself and give a little information on your family background, housing, and socioeconomic status? How did you know about your TB status and what was your reaction afterward? Can you share your experience with family members after testing positive? What were your reasons to seek medical attention? What type of medication was obtained? What is your general idea of TB? What is your view of the healthcare services provided and has TB affected your lifestyle?

Each participant was timed during the session (an average of 10 minutes for each participant) while ensuring data saturation before proceeding to the next participant. Data saturation was considered to be attained when no new information could be gleaned from the participants’ responses. Those requiring more time to reach data saturation were allocated an additional 3 minutes to wrap up the discussion. Overall, the session was completed within 2 hours. The session was audio-recorded with the approval of each participant as stated in the consent form.

### Qualitative analysis.

Data obtained from the FGD were analyzed using the NVivo 12 analysis software. A thematic analysis was performed in which aspects of a phenomenon described by the TB patients was analyzed in depth and checked for any interconnection. Thematic analysis was considered as the findings were mainly based on qualitative data and were not to be used for quantitative analysis. Themes and codes extracted from the FGD were explored and categorized based on the interrelationship of the information. The analysis was performed by four experts in thematic analysis. All the experts discussed and agreed on the emerging themes and interrelated responses.

## RESULTS

### Descriptive results.

The FGD was conducted among 10 TB patients who had already completed their compulsory 6-month treatment course at various district clinics in Selangor. All the participants provided signed and oral consent and completed the FGD session. Table 1 presents demographic information of the 10 participants. The participants comprised seven males (P1, P4, P5, P6, P7, P8, and P10) and three females (P2, P3, and P9), but all were Malays and Muslims. Eight of them had a formal education, except P1 and P10. Four participants were government servants (P1, P2, P7, and P10), two were self-employed (P3 and P9), one was a chef (P4), and one was restaurant staff (P6). They all stayed in a location with a moderate to high number of foreigners.

**Table 1:** Demographic information of TB patients who participated in the focus group discussion and in-depth interview

Variable	P1	P2	P3	P4	P5	P6	P7	P8	P9	P10
Sex	Male	Female	Female	Male	Male	Male	Male	Male	Female	Male
Education	None	Formal	Formal	Formal (SPM)	Formal	Form 4	Formal	Formal	Formal	None
Occupation	Government Servant	Government Servant	Self-Employed	Chef	None (retired)	Restaurant (dependent)	Government Servant	N/A	Self-Employed	Government Servant
Race	Malay	Malay	Malay	Malay	Malay	Malay	Malay	Malay	Malay	Malay
Religion	Islam	Islam	Islam	Islam	Islam	Islam	Islam	Islam	Islam	Islam
Type of House	N/A	Own House	Rent	Flat and Renting	Rent	Own Property	Rent	N/A	Rent	Own House
Presence of Foreigners	Yes	Yes	Yes	Yes	Yes	Yes	Yes	Yes	Yes	None
Number in Household	5	N/A	7	N/A	3	18	6	8	11	5
Insurance	N/A	N/A	N/A	N/A	None	N/A	N/A	N/A	None	N/A
Smoking	Yes	No	No	Yes	Yes	Yes	Yes	No	No	Yes
Comorbidity	High Blood Pressure	N/A	N/A	Diabetes	High Blood Pressure	N/A	N/A	High Blood Pressure	Diabetes	N/A

N/A = not available; P = participant; SPM = Sijil Pelajaran Malaysia; TB = tuberculosis.

### Main emerging themes.

A total of 23 codes were generated from the thematic analysis of the FGD. These codes were further developed into seven main themes according to the objectives of the qualitative study, as shown in Table 2. These themes were as follows: health-seeking behavior, patient-related factors, healthcare-related factors, socioeconomic factors, knowledge of TB, challenges faced during the diagnosis and treatment of TB, and suggestions for preventing the delayed presentation of TB patients.

**Table 2:** Themes and codes developed via thematic analysis

No.	Themes	Subthemes and Codes
1	Health-Seeking Behavior	Postdiagnosis Reaction/Issues
		First Treatment Provider
		Perception of Healthcare Facilities
		Post-TB Life
		Satisfaction with Treatment
2	Patient- and Healthcare-Related Factors	Compliance
		Duration to Diagnosis
		Reasons for Delay
		Comorbidity
		Distance to the Health Facility
		Treatment Performed
3	Socioeconomic Factors	Funding Treatment
		Family and Social Support
4	Knowledge of TB	Knowledge of TB
5	Challenges Faced during the Diagnosis and Treatment of TB	Financial Constraint
		Postmedication Reactions
		Stigmatization
6	Suggestions for Preventing the Delayed Presentation of TB Patients	Working Environment
		Patient-Centered Care
		Clinical and Social Support
		Accessibility

No. = number; TB = tuberculosis.

### Health-seeking behavior.

The subthemes under health-seeking behavior comprised “postdiagnosis reaction/issues,” “post-treatment reaction,” “first treatment provider,” “perception of healthcare facilities,” “life after TB,” and “satisfaction with treatment.”

#### Postdiagnosis reaction/issues.

All participants shared their reactions immediately after they were diagnosed with TB. These reactions could be further classified into negative and positive reactions as reflected in the following statements.

“I did not face any problem at all, I was just afraid. On the day that I got to know that I was having TB, my kids were very sad and they did not sleep for the whole day. I had to wear a mask whenever I go out or visit my mum because I was scared of the infections.” (Participant 4)

Similarly, one of the TB patients elucidated her pessimistic mindset upon realizing she was positive for TB. In the words of the participant:

“I was down, shocked, and very angry because I was surprised to get infected with such a disease.” (Participant 5)

Meanwhile, Participants 2 and 6 exhibited a positive postdiagnosis reaction, which was probably linked to education about TB and support from family, as presented below:

“I knew I was about to get TB; I was ready for it.”

“First, I was shocked, then I started to think positive, my husband was very supportive and he mentioned that all diseases could be treated.” (Participant 2)

“I’ve never expected that I would contract such a serious disease, but then I realized that there are antidotes to cure. Then I started thinking about how to take care of myself and my work. Although I was not relaxed, I had to accept my fate.” (Participant 6)

Overall, the findings revealed that most TB patients were shocked upon knowing their positive status and were uncertain how they got infected. Although some patients were immediately optimistic about being treated, a few exhibited negative reactions that could eventually influence other events in their TB journey.

#### First treatment provider.

The government hospital or private clinic was the first health facility visited by most patients. Meanwhile, centers providing traditional therapy were later visited by patients when they faced issues related to delayed diagnosis or treatment. These events are highlighted in the following statements:

“I will go to Klinik Kesihatan first, then followed by a private clinic.” (Participant 5)

“I will first visit a modern hospital, but if they are unable to detect anything, I will go traditional.” (Participant 4)

“I was too tired even to walk. So I went to Taman Medan. The doctors there checked and diagnosed me with TB.” (Participant 7)

“I went to the government hospital first because the symptom that I had was fever, flu, and cough. So, I thought it was nothing.” (Participant 3)

#### Satisfaction with treatment.

Most patients were satisfied with the treatment received from the health facilities. The terms used to describe their satisfaction included “efficiency” and “good.” According to Participant 1, “I received treatment from Dr. Mega. It was very efficient.” Meanwhile, another patient opined that “I think the treatment can be improved, but the treatment I received was very efficient and good.”

#### Life after TB.

Given that some of the TB patients have been successfully cured, a subtheme was developed under health-seeking behavior titled “life after TB.” This aspect could be viewed as the degree to which the previous history of TB had influenced participants’ lifestyles and routine activities. As a result, the recurrent lifestyle activities in participants’ responses were “travelling,” “uncertainty about the cure of TB,” “smoking,” and “crowded environment.” Specifically, Participant 1 shared his view that previous exposure to TB had reduced his passion for traveling, as follows:

“I really love to travel, but maybe the bacterial infection that I contracted was because of my frequent travels; I have to limit this activity and be brave.” (Participant 1)

“In fact, I already stopped travelling. I am scared.” (Participant 2)

Another participant conveyed the impact of TB on his smoking behavior as depicted in the following statement:

“I feel better after being cured of TB because I have stopped smoking.” Yes, I used to smoke four packets in a day, now not even one.” (Participant 8)

### Patient- and health-related factors.

The subthemes developed were close contact, comorbidity, complaint, duration to diagnosis, and reasons for delayed presentation. A good number of the TB patients provided vital information about close contacts who may have contracted the disease from them, which included friends and family members. The following statements are the responses of P1, P4, and P5 when asked about their close contacts:

“Yes, my father. But he already passed away.” (Participant 1)

“My friend had TB of the bone.” (Participant 4)

“My friend from work. He had asthma and was coughing. I didn’t know that it was TB; it was just a normal cough.” (Participant 5)

“I brought my wife for examination because we are very close. I feel relieved because she is fine and not infected.” (Participant 4)

In terms of comorbidity, the common disorders mentioned by patients were high blood pressure and diabetes. Meanwhile, the prevalent symptoms were cough (with and without phlegm; all participants excluding P1), weight loss (all participants), difficulty breathing (P6), fever (P4 and P6), weakness (all participants), vomiting with blood (P1), body pain (P4), and chest pain (P3). Specifically, P2 stated that she lost 12 kg in 1 month. Meanwhile, the time taken by patients to reach the nearest health facility ranged from 10 to 45 minutes depending on the means of transportation.

The possible events that might have led to delayed presentation and diagnosis of TB among the participants were distance to a healthcare facility, awareness of the nearest health facility, the specific health facility visited or the first health personnel consulted, and poor health-seeking behavior (low suspicion index).

### Socioeconomic factors.

Factors relating to socioeconomic dimensions were derived from codes or subthemes relating to specific and combined sources of funds for treatment. Participants used various sources to fund their TB treatment, which included personal savings and financial support from religious members or organizations set up by the government. According to Participant 1, “I used my savings, which is finished now immediately after COVID-19. I was jobless for nearly 6–8 months; I was just sitting at home.*”*

On the other hand, Participants 5 and 3 posited that they received financial assistance from the Zakat (Annual Alms Tax) department and religious members, but the former still needed to support it by selling his personal belongings to proceed with his TB treatment.

“The Zakat department gave me some money because I was sick. Although the money is not really enough for me, the Surau members also contributed about RM 200 monthly. So overall, I had RM 600, but still, it was not enough for me, so I started to sell my unused things to get some money.” (Participant 5)

Although the financial assistance was also insufficient to cater to P3’s needs, the participant did not disclose her other sources of income.

“I am taking Zakat from the welfare department, and both amount to a sum of RM 900, but I have support from other means.” (Participant 3)

### Challenges faced after diagnosis and during treatment.

The challenges or barriers faced after TB diagnosis and in the course of treatment were conveyed by the patients, which can be categorized into the following: financial constraints, postmedication reactions, and stigmatization.

#### Financial constraints.

In terms of financial constraints, the patients mostly complained about the cost of medication and transportation, as typified in the statement below:

“The doctor asked me to eat medicines which were very expensive; they were like RM 200 per month.” (Participant 1)

Transportation issues were also relayed by some patients because they had to visit the healthcare facility using a commercial vehicle or those of their spouses when available. This event is reflected in the response from Participant 2:

“I faced several obstacles during my second treatment phase, especially transportation. My husband was working, so he would be the one to send me off to the hospital. I could not come every day because of this transportation issue.”

#### Postmedication reactions.

Meanwhile, postmedication reactions were reflected in the responses of most patients, as depicted in the following statements:

“I feel like vomiting, but the medicine is very important for TB.” (Participant 4)

“I kept eating the medicine, but I was vomiting after each round of medication.” (Participant 5)

Although some patients had to either abandon their jobs or had their salary reduced as they were unable to discharge their job responsibilities, others were still allowed to work in isolation. However, one of the participants did not reveal his TB status and kept working during treatment.

#### Stigmatization.

This subtheme could be considered an aspect of health-seeking behavior and the challenges faced upon being diagnosed and treated for TB. All the participants in this study shared diverse views relating to stigma either from their inner feelings or interactions with family members, friends, and immediate society.

The identified issues were related to not disclosing their TB status, isolation, and negative comments and actions by neighbors and community members. However, TB patients reacted differently to this issue, with some deciding to isolate themselves from their family and society, whereas others considered such reactions normal and moved on with their treatment.

Participants 1 and 4 disclosed their reasons for hiding their TB status as follows:

“The first reason why I didn’t disclose it was that I don’t want them to be afraid. Second, I don’t want them to be offended.” (Participant 1)

“I never revealed my sickness because if I did, everybody would stay away from me and I would be alone. This would be very difficult for me to bear.” (Participant 4)

Meanwhile, negative comments and actions, especially from neighbors and community members, were highlighted in the following responses:

“My sister supported me a lot, but her friends started saying that I wouldn’t be alive for so long. They were also saying that since I was using a stick, I wouldn’t be alive for long.” (Participant 1)

“Some of my neighbors tell their children or grandchildren that there are sick people in my house and that they should not play with my children.” (Participant 3)

Notably, only a single TB patient isolated herself based on the recommendations from the workplace. For fear of being stigmatized, the TB patients resorted to isolating themselves from society as depicted by Participants 5 and 6.

“I didn’t go out of my house so that people would not see me awkwardly. My mum told me to isolate myself, that’s all.” (Participant 5)

“Some people were aware of my TB status, while others were unaware. Since I live in a *Program Perumahan Rakyat* housing area, I always close my doors so that nobody will know anything.” (Participant 6)

### Knowledge and awareness of TB.

Thematic analyses provided insight into the patients’ knowledge of TB, such as the causes, symptoms, predisposing or risk factors, transmission or source of infection, and prevention and control. The first subtheme emphasized their general knowledge and awareness of the effects of TB on health. Diverse knowledge ranging from good to poor was demonstrated by the TB patients, as presented in the following statements:

“I never thought of the effects of this disease because the bacteria cannot be seen through naked eyes and it infects deep inside.” (Participant 4)

“I have not heard previously about TB, and I don’t know anything about the disease.” (Participant 6)

“I have heard before but I did not expect that I will be infected.” (Participant 3)

The next subtheme presents their knowledge of the symptoms of TB. Likewise, different perspectives were conveyed regarding the symptoms of TB. For instance, Participant 1 never thought of TB despite manifesting severe symptoms as reflected below:

“I never thought that I was infected with TB when I was coughing till bleeding.” (Participant 1)

This finding is in sharp contrast to the response of Participant 4 who suspected TB immediately after experiencing related symptoms such as coughing and body pain. This event was expressed in the participant’s response as follows:

“I was pretty confident that it was TB, especially after I started coughing, bleeding, and experiencing body pain. My friend went through all this, so I did not want to delay.” (Participant 4)

The third subtheme under participants’ knowledge of TB is potential transmission routes. Likewise, contrasting views were relayed as highlighted below:

“It cannot be transmitted by sneezing, coughing, or if not close our mouth.”

“I was afraid of my mother who is 60 years old and also my siblings. I didn’t want them to get infected. So I requested to use different utensils.” (Participant 1)

In terms of susceptibility and high risk of infection, all participants believed that TB could infect anybody irrespective of social class and educational level. This can be perceived from the following statements:

“This disease does not see whether the person belongs to the low or high class. High-class people are also infected.”

“This is an infectious disease, so it will definitely infect anybody no matter what.”

“Whether it is low or high, it seems everyone will get infected with TB; both mindsets are the same.” (Participants 1, 4, and 10)

Meanwhile, one participant shared his view about the risk of TB among smokers, alcohol consumers, and impoverished individuals as follows:

“I don’t smoke or drink alcohol, so how I can be infected with TB? I am a very poor person too, and I am not from a rich family. Although I attended crowded gatherings such as supermarket, Pasar Malam, and masjid, I accept it as my fate.” (Participant 5)

### Family and social support.

All participants living with their family members, excluding P5, highlighted the positive role played by the latter when managing their conditions. Most of the support stemmed from spouses, wives, and husbands, whereas a few mentioned their siblings and children. The supports included motivation, care, prayer, and assistance during routine activities in the health facility. In the words of P1, P2, and P3:

“I have to think about this because it is an infectious disease, but my husband is very caring. I initially wanted to isolate myself in a different room, but my husband did not let me. He was ready to be infected in the process of caring for me. He also mentioned that we are married and will always stick with me irrespective of my health condition. He even wanted to take leave without pay to take care of me for a month.” (Participant 2)

“My family supported me a lot with motivation; we prayed together several times to fight the disease. I was initially isolating myself because I know that the disease is infectious.” (Participant 3)

“My wife supported me a lot, but her friends started saying that I won’t be alive for long.” (Participant 1)

Although the wife of P5 was supportive and caring, the TB patient elaborated on the bad experience with his child during his TB journey. Meanwhile, one participant was not staying with family members when he was diagnosed with TB; he had to support himself and received no assistance from friends despite staying in adjacent rooms. This can be gleaned from the following responses:

“I am staying together with my wife and eldest child. He has never given me a single cent. My eldest child has been living with me for quite a long time. I have been tolerating him for a long time, but I don’t mind about it at all.” (Participant 5)

“I was supporting myself. Although my friends were aware that I was infected with TB, I did not receive any support from them since everyone stayed in different rooms.” (Participant 5)

### Suggestions for preventing TB infection and delayed presentation.

Suggestions to prevent delay in seeking treatment among TB patients were gleaned from the FGD, which were mainly categorized into operation and organization of TB health facilities, education and awareness, self-care, clinical and social support, and accessibility to healthcare facilities.

In terms of the operation and organization of healthcare facilities and centers, the areas that need to be improved include the working environment, infrastructure, and operational management. The participants advocated that the interplay of these factors would make them feel comfortable in seeking treatment at public TB centers. One of the participants shared her experience as follows:

“This TB clinic needs to be upgraded; I don’t even want to go there. The place is horrible, that housing at the side, cramped with cars, motor parking. It’s not convenient at all as a hospital. Terrible place.”

“More chairs are needed in the waiting area. Yes, comfortable facilities, and as mentioned earlier, try not to separate TB patients from others.” (Participant 4)

Educating people to enhance their awareness of TB was highlighted by most participants as crucial in reducing problems associated with visiting appropriate TB centers or nearest health facilities. This issue was conveyed by participants by comparing the perceived knowledge of TB and other well-known diseases affecting Malaysians. This view was shared by P1 as follows:

“For AIDS, it has been written everywhere but TB nothing. Many people don’t know this disease, because it does not spread like AIDS.” (Participant 1)

Another perspective relayed by participants was self-care and management, which comprised the consumption of healthy and nutritious food and lifestyle activities. Clinical and social support entailed suggestions on the provision of home test kits for rapid detection of TB and patient-centered care during consultation and treatment. An example of this view is highlighted in the following response:

“Would be better if test kits are provided for us to identify the disease earlier.” (Participant 6)

Participants also disclosed the need to improve accessibility to healthcare facilities. The main factors mentioned included creating specific clinics for TB patients and proximity of health facilities to facilitate a prompt response, minimize cost, increase the capacity to isolate infected persons, and provide adequate treatment. The following statements elucidate the points mentioned above:

“Better to have nearer clinics, don’t have to build far away. Somewhere near the community. Don’t have to spend up to RM 20. For me, I feel like Ministry of Health (MOH) should have separate clinics for TB patients.” (Participant 2)

“The registration places are similar to others, which is advisable to make it in different places.” (Participant 4)

## DISCUSSION

This study comprised a combination of FGD and in-depth interviews to understand patients’ perspectives of events surrounding delayed presentation and diagnosis of TB. Several factors contribute to the delayed presentation of TB, which varies significantly in different populations and healthcare settings. Given the high burden of TB in Selangor, this study provides first-hand evidence from TB patients to elucidate the barriers and facilitators of seeking medical attention for TB diagnosis and treatment, thereby assisting in designing locally tailored interventions to address patient and hospital delays.

Descriptively, most of the participants in the survey were males and had formal education, which is consistent with a former cross-sectional study on the prevalence and risk factors for TB in Malaysia.^16^^,^^17^ All the participants were Muslims and Malays, which equally reflects the demographic distribution of the Malaysian population. Six of the TB patients had a history of smoking, either current smokers or having quit smoking. Similarly, Goroh et al.^16^ found that one-third of TB patients were smokers based on the trend from 2012 to 2018.

The first theme that emerged from the thematic analysis was health-seeking behaviors, which comprised post-treatment reaction, first treatment provider, perception of healthcare facilities, life after TB, and satisfaction with treatment. Participants shared both negative and positive reactions upon knowing their positive TB status. Tuberculosis patients reacted differently upon being diagnosed or informed about their positive status, which may be linked to their prior perception and awareness of the disease. Those reacting positively immediately after diagnosis might be aware of possible sources of exposure to the pathogen or know that the disease is treatable.^23^^–^^26^ Nevertheless, the diverse reactions reflect the need for proper education and enlightening patients regarding the importance of prompt detection and treatment. In addition, community awareness programs could be implemented to raise people’s understanding of TB.

Meanwhile, government hospitals or private clinics were the first health facilities visited by most TB patients, which corroborates the results in previous local studies.^7^^,^^16^ Accessibility and previous experience with these healthcare providers might contribute to this finding as documented in studies conducted elsewhere.^27^ Related closely to this finding was overall patient satisfaction with the treatment received from the health facilities. The TB patients described the treatment received either as efficient or good, which could stem from the fact that they had already completed their 6-month treatment course for TB. The good outcome and absence of clinical manifestation of the disease might contribute to patients’ perception of the treatment received.

A subtheme termed “life after TB” was also gleaned from the thematic analysis under the theme of health-seeking behavior. The findings demonstrated the impact of TB diagnosis and treatment on patient lifestyle and behaviors. Some of the participants had already quit smoking, whereas others had stopped touring different countries after their TB journey. This result aligns with the concept of the Health Believe Model, whereby the cue to action is influenced by perceived susceptibility, perceived seriousness, perceived benefits, perceived barriers, perceived threats, and self-efficiency.^21^ Tuberculosis patients in this study understood the perceived susceptibility and perceived threats associated with the disease, thereby influencing their cue to actions in engaging in lifestyle or activities that might increase their risk of exposure.

The second theme identified in this study comprised patient and healthcare-related factors, as well as events contributing to delayed presentation and diagnosis of TB, composed of distance to a healthcare facility, awareness of the nearest health facility, the specific health facility visited or the first health personnel consulted, and poor health-seeking behavior (low suspicion index). These results indicate that distance and time spent before seeing a consultant or clinician influenced patients’ decisions to visit TB care centers or healthcare facilities, as reported in studies conducted in other countries.^23^^,^^27^^–^^29^ Having to move a long distance before reaching a hospital or clinic is time-consuming and not cost friendly, thereby discouraging TB patients from seeking treatment and leading to late presentation.

Prolonged waiting time at directly observed treatment was also a predictor of early treatment interruption in a recent study undertaken in Malaysia among TB patients.^30^^,^^31^ Likewise, the factors reported to influence the delayed presentation of TB patients in Sabah were poor transportation systems and challenging topography,^16^ which align with the present results. Notwithstanding, awareness of the availability of such facilities, specifically TB care centers, is equally important for individuals in order to seek prompt diagnosis and treatment. Overall, these events need to be supported with good health-seeking behaviors with an acceptable suspicion index.^25^

The present study regarding delayed presentation has important implications for the management of TB patients in Selangor. The likelihood of achieving a positive immune response and successful management is higher when TB is detected early, followed by immediate treatment. Meanwhile, patients delaying presentation to healthcare centers for 1 month or more are possibly at advanced stages of the disease and manifesting severe symptoms.^32^ These events will increase the burden on existing healthcare facilities given that patients may have to be admitted for advanced care or subjected to more intensive management. In addition, the economic burden on patients and the healthcare system increases accordingly, especially when patients are responsible for their treatment expenditures.^6^

Tuberculosis patients in this study relayed the difficulties faced in managing their diseased status given their low socioeconomic status. Most complained that financial assistance was insufficient to cater to their needs upon contracting the disease. These factors were also identified in previous studies, as household income and financial insecurities were positively associated with the prevalence of patient delay and poor adherence to treatment protocol.^23^ Patients facing socioeconomic challenges are more likely to delay visits to healthcare centers given that they must concurrently cater to their families and healthcare expenditures despite having limited sources of income.

Furthermore, we observed that low socioeconomic status, financial constraints, and perceived stigmatization were among the key challenges faced by patients with TB. Tuberculosis patients used various sources of funds for treatment, comprising personal savings and financial support from religious members or organizations set up by the government. Nevertheless, most TB patients still needed financial assistance to support their current primary sources of income. Although a clear role of financial constraints can be gleaned from this study, the issue of stigmatization as a potential challenge to seeking prompt care and diagnosing the disease requires further clarification. The participants in the FGD shared diverse views relating to stigma either from their inner feelings or interaction with family members, friends, and immediate society. Further analysis reflects that the socioeconomic status of the patients seemed to shape their opinion and the perceived stigmatization they might face in their immediate household and community. For instance, the few respondents who delayed seeking care and remained isolated in their homes were relatively from a low socioeconomic class. Similar events were raised by TB patients responding to the surveys conducted in Nigeria^24^ and Sudan.^25^ More importantly, these studies reflected positive associations between longer patient delay and TB patients reporting perceived stigma. On the other hand, a few respondents decided to stay indoors in line with the recommendations from healthcare professionals and the workplace. Despite highlighting the stigma they might have encountered if they continued to go to work, such perceived stigma did not translate to delayed treatment or diagnosis of the disease among this group of respondents. Previous studies have also highlighted how perceived stigma may be influenced by interaction between healthcare providers and TB patients.^16^^,^^26^ Nevertheless, there are no reports to infer such events in the present study, as no respondent linked their perceived stigma to their engagement with healthcare workers.

Another key finding in this study was the diverse knowledge of TB in terms of the causes, risk factors, and treatment and their association with delayed presentation. For instance, although some patients were aware of the symptoms, others posited that they had never heard of the disease and were unaware that cough is a common sign of TB. Such poor knowledge persisted even after treatment, as some patients even doubted the cure of TB during treatment. Alarmingly, one of the participants claimed “not to be aware of being infected with TB even while coughing with blood.” These findings are supported by a recent study conducted in Sabah in which poor patient knowledge influenced the delayed presentation of TB patients to healthcare facilities.^16^

In contrast, another participant exhibited a high suspicion index by reporting immediately upon experiencing cough and body pain. Poor knowledge of TB has also been reported in previous studies to increase the odds of patient delay.^23^^–^^26^ Patients who are fully aware of the symptoms and consequences of late presentation and delayed treatment are more likely to visit healthcare centers for diagnosis and management. Such patients will thus avoid any delay in seeking treatment at nearby hospitals. However, some patients with good knowledge of TB symptoms and medication might also engage in self-management, which could result in delayed presentation.^26^

The last theme focused on suggestions to address the factors contributing to delayed treatment of TB patients. For instance, patients also gauged their current knowledge of TB and suggested that educating people to enhance their awareness of the disease is crucial in reducing problems associated with visiting appropriate TB centers or the nearest health facilities and with delayed presentation. These recommendations coincide with the conclusions drawn from prior studies in which educating high-risk populations of TB was seen as the most practical and feasible approach to addressing patient and treatment delays.^26^^–^^28^

Our findings revealed the importance of family and social support during TB management, particularly from patients’ spouses, siblings, and children in the form of motivation, care, prayer, and assistance during routine activities in the health facility. These events formed a strong support system that was vital in combating issues relating to stigma and harassment either from family members or the community. Such events will ultimately encourage individuals to consult the appropriate centers for diagnosis and treatment if necessary. Social support from family members and the immediate community has been shown to positively impact compliance and treatment and enhance the psychological well-being of TB patients.^29^ On the other hand, poor compliance with TB therapy is associated with drug resistance, chronic infection, and mortality.^30^ Support from the family and community is also vital in addressing the stigma of TB—one of the key factors contributing to delayed presentation and diagnosis. The present finding coincides with that of Goroh et al.^16^ who found that stigmatization of TB patients was a strong determinant of patient delay in the Borneo region of Malaysia

Other important suggestions highlighted by TB patients during the FGD include improving accessibility to healthcare facilities, providing specific clinics, and proximity of health facilities to facilitate a prompt response, minimize cost, increase the capacity to isolate infected persons and provide adequate treatment. These recommendations represent the opinions of the respondents; however, the feasibility of implementing some of the measures remains debatable. For instance, establishing specialized TB clinics may constitute a costly approach given the material and human resources required. Moreover, there are existing centers designed for the management of infectious diseases in all government hospitals in Malaysia. There are no clear-cut advantages of establishing specialized centers in terms of the management of TB. In addition, attempting such will also necessitate creating specialized centers for other infectious diseases in the country.

Thus, further investigation is required to understand what might have informed respondents to make such recommendations. Perceived stigma or feeling embarrassed during the interaction with healthcare providers may equally contribute to the respondents’ view of establishing specialized centers. This is plausible given that all visiting patients would be there for the same purpose and healthcare workers would be specifically trained for disease management. Nevertheless, because having specialized centers will not in any way alter the community’s perception of the disease, approaches such as education and awareness remain the most feasible and effective approach to addressing the issue of stigma.^31^ These recommendations align with the findings of Suliman et al.,^31^ who investigated the determinants of early treatment obstruction among TB patients in Malaysia. The researchers disclosed that long waiting times and short-course centers increased the odds of early treatment interruption in newly diagnosed patients. Experiencing long waits remains a burden for patients as they had to take frequent medical leave from their jobs and lost income needed for living expenses, thereby affecting the treatment plan. In addition, longer waiting times diminish patients’ satisfaction with services rendered by health systems, leading to a high dropout rate from treatment.^31^

Similar events apply to delayed presentation given that factors such as inaccessibility and handling of all stages of TB cases under the same setting will result in long waiting times and diagnosis delays. In terms of the operation and organization of healthcare facilities and centers, participants also mentioned areas requiring improvements, such as the working environment, infrastructure, and operational management. Notably, the participants noted that the interplay of these factors would make them feel comfortable in seeking treatment at public TB centers. Hence, the suggestions might be considered by policymakers and healthcare providers to encourage patients to engage in the early presentation of suspected TB cases.

## CONCLUSION

This study revealed that patient- and healthcare-related factors, health-seeking behavior, socioeconomic status, and knowledge of TB were pertinent events that may shape the barriers and facilitators of ensuring prompt presentation and diagnosis of TB patients in Selangor. Specifically, problems such as financial constraints, inaccessibility and long distance to healthcare facilities, poor knowledge of TB, and lack of family and social support contributed to delayed TB presentation and diagnosis. Meanwhile, the role of perceived stigma in shaping patients’ decisions and behavior toward diagnosis and treatment requires further investigation. Apart from addressing the aforementioned issues, additional suggested interventions included establishing specific clinics and proximity of health facilities to facilitate a prompt response, increasing the capacity to isolate infected persons, and providing adequate treatment. These findings may be considered in the development of effective, locally tailored interventions to address delayed presentation and diagnosis of TB patients in Selangor.

Given the present research limitations, a longitudinal and prospective study could be attempted in future research to better understand the sequence of events leading to delayed presentation among TB patients. Apart from that, interventions to prevent patient and hospital delays could also be developed based on the present findings and reports in the previous literature. Qualitative studies such as interviews or FGDs need to be undertaken with a greater number of healthcare practitioners, particularly specialists and those working in TB centers and healthcare facilities receiving a high volume of TB patients. This is highly recommended, as the research instrument used in this study focused more on patient delay than on hospital and treatment delay. Future research may be expanded to other states with a high TB burden in Malaysia.
